# Research unit network (RUN) as a learning research system

**DOI:** 10.1017/cts.2023.514

**Published:** 2023-03-27

**Authors:** Alejandro P. Comellas, Jackline M. Wangui-Verry, Kimberly J. Sprenger, Patricia L. Winokur, Patrick B. Barlow, Maran Subramain

**Affiliations:** 1 Institute for Clinical and Translational Science, University of Iowa, Iowa City, IA, USA; 2 Department of Internal Medicine, University of Iowa, Iowa City, IA, USA

**Keywords:** CTSAtsa, RUN, research unit network, clinical research units, learning research system

## Abstract

The clinical research units (CRUs) are one of the main spaces where both translational research and science take place. However, there is a lack of information about both best practices for CRU operations and, ultimately, benchmarks to evaluate CRU performance. The Research Unit Network (RUN) was created with the purpose to enable direct communication and collaboration among CRUs. An online survey was administered to further illustrate the functionality and impact of RUN. Thirty-one individual survey responses (39.2%) were included in the final analysis. The members value RUN monthly meetings (87.1%) as the most useful aspect of this network and CRU budgeting (67.7%) and staffing (61.3%) were the most relevant topics discussed. This is followed by EPIC – Research (58.1%), delegation of authority logs, unit signatures, and policies (51.6%), COVID-19 pandemic response (41.9%), the implementation of clinical trial management system (29.0%), and protocol deviations (19.4%). The intermediate goal of RUN is to identify best practices CRUs are establishing, implementing, and sharing these experiences with the goal to adopt them in different CRUs. The network’s long-term goal is to establish standard benchmarks that can be used for evaluating the performance of CRUs across the nation.

## Introduction

Clinical and translational research follows specific standards and protocols. Many of these trials are conducted in clinical research units (CRUs). However, there is a lack of information about both best practices for CRUs operations and, ultimately, benchmarks to evaluate CRUs performance.

### Research Unit Network as a Learning Research System

Translational science is the field which studies the translational process in order to establish governing scientific principles with the goal of leading increases in productivity, efficiency, and capability [1]. The National Center for Advancing Translational Sciences (NCATS) has placed a strong focus on translational science with the goal of improving common causes of inefficiencies and failures in translational research projects. This manuscript is an introduction to the Research Unit Network (RUN), and how the network is evaluating CRUs around the country, to begin to understand best practices and develop a learning platform for enhancing efficiencies in CRUs.

### Research Unit Network

**Fig. 1.**
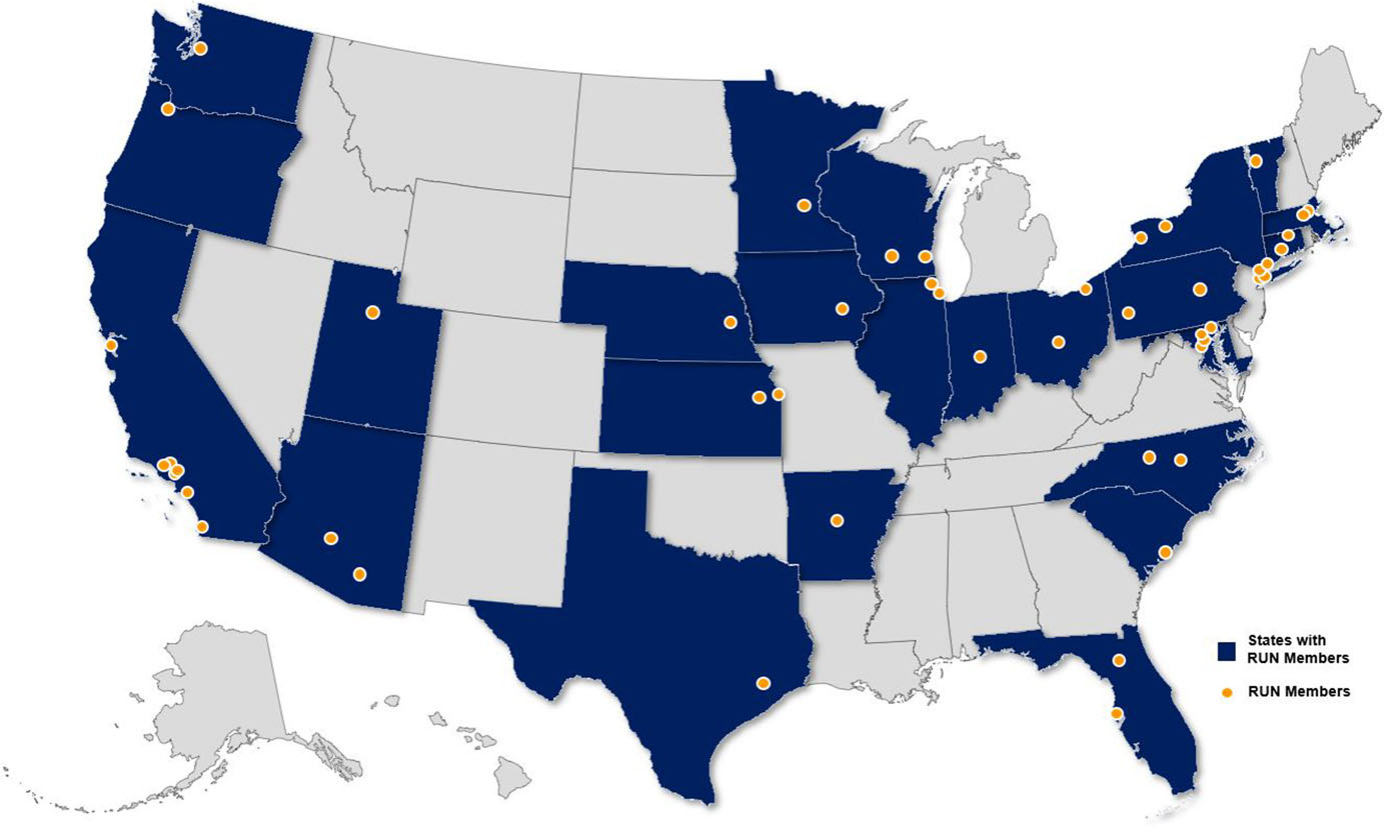
Research Unit Network (RUN) Members Institutes (as of June 2022).

The CRUs are one of the main spaces where both translational research and science take place. In 2014, NCATS the National Institutes of Health (NIH) announced that direct support of the CRUs would no longer be allowed for any CTSA, leading to the need for hospitals and other research institutes to operate these entities on a service center model [2]. This decision resulted in overlooking these units from any discussion related to translational science. To re-establish dialog between CRUs that would help units benchmark and improve operations, RUN was created with the express purpose of enabling direct communication, sharing, and collaboration among CRUs. RUN held its first meeting on July 25, 2018, led by the University of Iowa. At the time, eight institutions joined the network. Within 4 years, the network has grown to 50 institutions, with 79 members. Forty-two CRUs are associated with institutions that are funded by NIH, NCATS Clinical Translational Science Awards (CTSA), six of the CRUs are located in institutions supported by NIH, NIGMS through the Institutional Development Award Networks for Clinical and Translational Research, and two other CRUs have joined since (Fig. 1). The network provides the opportunity to discuss topics relevant to the operations of clinical and translational research, with the goal of improving translational science. The discussions take place on monthly video calls and on the NCATS Center for Leading Innovation & Collaboration (CLIC) online discussion forum. Some of the topics discussed include: Development and implementation of standard operating procedures (SOPs) at CRUs; Delegation of authority logs; Electronic orders and source documents; and Workforce Development.

Other areas discussed in RUN include: Processes to request services in CRUs; Recruitment and retention of CRU workforce; Pediatric research in CRUs; General budget guidelines; CRU price list; Calibration and equipment maintenance; Verification of doses; Sponsor attendance during study visits; Personal protective equipment for investigational drugs in context of USP800 requirements; and Recruiting special populations, including under-represented minority participants.

In addition to facilitating discussions around the topics mentioned already, RUN was pivotal in exchanging information and practices during the beginning of the COVID-19 pandemic [3]. This experience resulted in the identification of the unique roles played by CRUs as part of a disaster response.

### Best Practices in CRUs

Nationally, there is a lack of information about both best practices for CRU operations and, ultimately, benchmarks to evaluate CRU performance. Benchmarking against other CRUs can provide input in areas that units are performing well in addition to highlighting areas where improvement is needed. To develop these benchmarks, it is important to have uniform agreement regarding best practices, which refers to prescriptive and aspirational norms that will result in increased efficiency and effectiveness of CRUs.

The short and intermediate goal of RUN is to identify best practices and to evaluate the process of change management that units have used to implement new standards. Ultimately, the goal is to use RUN to disseminate knowledge, create a collaborative national team that sites can utilize to improve management within their own institution, and serve as a body that is continuously evolving best practices as new clinical and translational research practices are needed (e.g., accommodation of remote monitoring during the COVID-19 pandemic). Through this team of national research professionals, RUN allows institutions to benchmark their own practices and presents a platform for systematic study of performance around the nation.

To advance this national benchmarking, RUN began two projects. First, evaluation of CRUs re-charge strategies as part of a Market Fairness Analysis. RUN is currently conducting a survey on research service changes among its members. Another project is the development of a protocol deviation survey at a RUN institution to identify areas of improvement to better support clinical trials.

### RUN as a Learning Research System – Members Feedback Survey

***RUN Members Feedback Survey:*** An online survey was administered to further illustrate the functionality and impact of RUN. Members (79) from all 50 RUN member institutes were invited to complete a brief online survey that was comprised of five questions focused on the most useful aspect of RUN (i.e., RUN monthly meeting, CLIC discussion forum); the most relevant topics discussed (i.e., CRU budgeting, CRU staffing, EPIC – research); the impact and importance of RUN to its members and their organizations; and suggestions on RUN’s future direction. The web-based survey was administered using the Qualtrics software, between September 21–29, 2022.

Thirty-one individual survey responses (39.2%) from 24 RUN member institutes were included in the final analysis. Descriptive data analyses were used to organize the information gathered via the online survey. Open and axial coding were used to identify general categories of information obtained from the open-ended survey responses and to sort those categories into related and meaningful groups.

***Data Analysis***, as illustrated in Table 1, the members value RUN monthly meetings (87.1%) as the most useful aspect of this network. This is followed by the CLIC Discussion Forum (interaction & resources) (51.6%) and Guidelines (SOPs) Development (41.9%). Table 2 shows the results of the discussion topics that were most relevant to RUN members and their organizations. According to RUN members, CRU budgeting (67.7%) and CRU staffing (61.3%) were the most relevant topics discussed. This is followed by EPIC – Research (58.1%), delegation of authority logs, unit signatures, and policies (51.6%), COVID-19 pandemic response (41.9%), the implementation of clinical trial management system (CTMS) (29.0%), protocol deviations (19.4%), and other topics (9.7%).

**Table 1.** Most useful aspect(s) of RUN for members

Response options	Frequency (%)
RUN monthly meeting	27 (87.1%)
Guidelines (SOPs) development	16 (51.6%)
The CLIC (email) discussion forum	13 (41.9%)
Other	6 (19.4%)

Abbreviations: RUN, Research Unit Network; SOPs, standard operating procedures.

*Note*: Participants could select multiple response options.

**Table 2.** Most relevant topic(s) discussed

Response options	Frequency (%)
CRU budgeting	21 (67.7%)
CRU staffing	19 (61.3%)
EPIC – research	18 (58.1%)
Delegation of authority logs, unit signatures, and policies	16 (51.6%)
COVID-19 pandemic response	13 (41.9%)
Implementation of CTMS	9 (29.0%)
Protocol deviations	6 (19.4%)
Other	3 (9.7%)

Abbreviations: CRU, clinical research unit; CTMS, clinical trial management system.

*Note*: Participants could select multiple response options.

***Qualitative Responses:*** General categories of information contained in the open-ended survey question responses were identified using open coding. Then, tentative labels were organized into related and meaningful groups of data using axial coding, which further assisted authors in refining, aligning, and categorizing the data into the five distinct themes. Authors further elaborated themes observed in the following section.

**Networking & Communication:** Primarily, RUN members indicated that the network is a place for them to collaborate and stay informed on current developments that are happening in CRUs across the country. The members mentioned that participating in RUN makes them feel they are a part of a network that is important and significant. Also, as a learning research system the ability to network provides the members and organizations with the opportunity to improve performance over time. Furthermore, the regular monthly meetings create a venue to discuss operational aspects of CRUs where all its members can exchange experiences with lessons learned that are shared within the network. This interaction results in implementation of practices that have a goal to increase efficiency and effectiveness of the CRUs in providing services to the research communities.

**Challenges & Best Practices:** Through RUN, members are able to share challenges related to operations in CRUs. These include development of best practices, recruitment, and retention of CRU workforce, how to prepare, respond, and recover from disasters such as the recent COVID-19 pandemic, including the development of strategies to maintain the units open and continue to provide services to essential research projects such as therapeutic trials. In addition, the network creates an environment where all learn from each other unique local regulations, which can impact some of the solutions, and recognizing the challenges in having universal approaches to same problems.

**Resource: Education and Improvement:** With RUN monthly meetings, a series of topics that are aligned with the unmet needs of CRUs around the nation were developed. This provides an opportunity to ask questions, share lessons learned and the development of new ideas that can improve processes. Furthermore, with the current CLIC forum site, we can store all presentations and supported literature as a resource for RUN members. Most importantly, RUN is very helpful to new CRUs and staff members. This attests by the following survey responses: “being a relatively new unit, RUN has been helpful in guiding me in the [CRU] processes,” and “we are just starting out and all the information and experience is very helpful, especially the budget.”

**Workforce and Budget:** Budget of CRUs is aligned with their services. Since many units have different services, capacities, and cost of living, the network provides an opportunity to share ideas about the average costs of similar services and also identify the unique services of each unit. Furthermore, aligning the workforce needs with the costs of services provides the opportunity to create instruments that are shared within RUN to justify some of the different requests that units have to present to administrators and leaders of their institutions. The most experienced RUN members are very valuable resources for all CRUs. Also, as members could potentially move to other cities/states, RUN becomes a networking resource to find possible job opportunities in the field of clinical and translational research.

**Standard Operating Procedures:** As one of the major goals of RUN is to develop benchmarks for clinical research, the development of SOPs is the tool to have best practices that can be implemented and evaluated across units. RUN is currently developing one new guideline per meeting, which is presented to the group for feedback and is finalized prior to the next meeting and stored in the RUN forum (https://app.ctsa.io/df?df=https://sites.google.com/ctsa.io/run).

## Discussion

### RUN as a Learning Research System

A learning health system is defined by the Institute of Medicine as a system in which science, informatics, incentives, and culture are aligned for continuous improvement and innovation, with best practices seamlessly embedded in the delivery process and new knowledge captured as an integral byproduct of the delivery experience [4]. In addition, Dr. Christopher Austin – (former) Director of NCATS, in a review on opportunities and challenges in translational science, proposes that improved effectiveness in translational science must be broadly and intentionally disseminated to enable the entire research community to apply them in their own translational research, which NCATS formalized as the “3Ds”: Develop, Demonstrate, and Disseminate [1]. We submit that RUN is fulfilling these 3Ds and work as a “learning research system,” where well-designed experiments to improve translational science can be developed, tested, implemented, and disseminated throughout different geographies and unit types.

In addition to the overwhelmingly positive survey responses, some RUN members also mentioned that “this is the only organization that speaks directly to my issues as manager of a CRU.” Additionally, RUN is also resourceful in accelerating members' learning about CRU operations. Collectively, RUN members recognize the need for and importance of training the workforce of newer clinical research institutes and are more than willing to share their wisdom and experiences, functioning as potential mentors. According to Hawkins & Fontenot [5], “the key to the development of leaders for the healthcare professions is mentoring.” As stated by Burgess *et al.* [6], “the importance of mentorship within health care training is well recognized and it offers a means to further enhance workforce performance and engagement, promote learning opportunities, and encourage multidisciplinary collaboration.” Nevertheless, this sharing of knowledge and mentoring experiences are not only benefitting the mentees (newer CRU members) but also mentors (seasoned CRU members), as it keeps everyone engaged in new developments of clinical research and the healthcare field in general.

Staffing and research budgets are among the two main challenges faced by majority of clinical research sites [7]. Additional and continuous guidance on staffing and research budgeting are also requested by RUN members. Thus, the upcoming RUN meetings and CLIC forum discussions are designed to address these concerns.

As stated earlier, the ultimate goal of RUN is to disseminate knowledge, create a collaborative national platform that sites can utilize to improve management within their own institution and serve as a body that is continuously evolving best practices as new clinical and translational research practices are needed. The RUN Members Feedback Survey clarifies that the network has already achieved these goals and can certainly grow into a more impactful and significant network with the continuous support from NCATS and similar entities.

In accordance, the “National CTSA Steering Committee meetings provided an opportunity to share best practices and develop the idea of capturing the CTSA program experiences in a series of papers” [8]. They have documented “creative innovations developed in response to the COVID-19 pandemic…for adoption as new standards, thus converting the painful trauma of the pandemic into “post-traumatic growth” that makes the clinical research enterprise stronger, more resilient, and more effective” [8]. In the same vein, RUN has also captured CRUs best practices during the COVID pandemic. Additionally, RUN is continuing the development and assessment of best practices among CRU members. Echoing NCATS mission, RUN initiatives are making the CRUs in the network stronger, more resilient, and more effective.

Drs David G. Nathan and David M. Nathan entitled their opinion editorial as “Eulogy for the clinical research center” [2]. They mourned the loss of the CRUs (NIH extramural funding) as they acknowledge their historical contributions and the incredible value of the human resources in these units. Although we agree with their assessment of the CRU value, RUN has demonstrated that these units are vibrant and continue to fulfill the mission of advancing clinical and translational research. A network which was practically created with a zero budget and purely on the interest of improving the efficiency of CRUs is currently thriving both with its membership and the studies being conducted. RUN now provides a unique opportunity to advance the mission of NCATS in studying the general principles of translational science from an empirical approach.

## Limitations

This study has several limitations which include a small sample size for gathering sufficient data that is representative and generalizable to all RUN members and CRUs across the USA. The collection and integration of other longitudinal and additional relevant data from current and new RUN members on a continuous basis are expected to provide richer perspectives and a more holistic view of the contributions of RUN to its members.

## Supplementary material

For supplementary material accompanying this paper visit https://doi.org/10.1017/cts.2023.514. click here to view supplementary material
