# Supplementary material for: Research unit network (RUN) as a learning research system
Source: J Clin Transl Sci. 2023 Mar 27;7(1):e89. doi: 10.1017/cts.2023.514 (PMC10130846; doi:10.1017/cts.2023.514)
Supplement: Supplementary file 1 [file S2059866123005149sup001.docx]

Supplementary Online Content

eTable 1. Research Unit Network (RUN) Members Institutes (as of June 2022)

**eTable 1. Research Unit Network (RUN) Members Institutes (as of June 2022)**

| - Arizona State University |
| --- |
| - University of Arizona |
| - University of Arkansas for Medical Sciences |
| - Cedars-Sinai Hospital – Los Angeles, CA |
| - Charles R. Drew University of Medicine and Science – Los Angeles, CA |
| - University of California, Irvine |
| - University of California, Los Angeles |
| - University of California, Lundquist Institute, Torrance |
| - University of California, San Diego |
| - University of California, San Francisco |
| - University of Connecticut / UConn Health |
| - Yale Center for Clinical Investigations / YNHH |
| - Children’s National – Washington, DC |
| - Georgetown University Medical Center |
| - University of Florida |
| - Johns Hopkins All Children’s Hospital |
| - Northwestern University Clinical and Translational Sciences |
| - University of Illinois, Chicago |
| - Indiana University |
| - University of Iowa |
| - The University of Kansas Medical Center |
| - University of Kansas |
| - Johns Hopkins Medicine International |
| - University of Maryland |
| - Boston Children’s Hospital (Harvard) |
| - Massachusetts General Hospital |
| - University of Minnesota |
| - University of Nebraska |
| - Columbia University |
| - Einstein and Montefiore |
| - New York Presbyterian Hospital Weill Cornell Medical Center |
| - NYU Langone Health |
| - University of Buffalo |
| - University of Rochester Medical Center |
| - University of North Carolina at Chapel Hill |
| - Wake Forest |
| - Cleveland Clinic |
| - The Ohio State University |
| - Oregon Health and Science University |
| - Penn State |
| - University of Pittsburgh Medical Center |
| - Medical University of South Carolina |
| - University of Texas Health Science Center at Houston |
| - University of Utah Health |
| - The University of Vermont Medical Center |
| - University of Washington at Seattle |
| - Medical College of Wisconsin |
| - UW Health – University Wisconsin Hospital |
